# Influenza virus replication in cardiomyocytes drives heart dysfunction and fibrosis

**DOI:** 10.1126/sciadv.abm5371

**Published:** 2022-05-11

**Authors:** Adam D. Kenney, Stephanie L. Aron, Clara Gilbert, Naresh Kumar, Peng Chen, Adrian Eddy, Lizhi Zhang, Ashley Zani, Nahara Vargas-Maldonado, Samuel Speaks, Jeffrey Kawahara, Parker J. Denz, Lisa Dorn, Federica Accornero, Jianjie Ma, Hua Zhu, Murugesan V. S. Rajaram, Chuanxi Cai, Ryan A. Langlois, Jacob S. Yount

**Affiliations:** 1 Department of Microbial Infection and Immunity, The Ohio State University, Columbus, OH, USA.; 2 Infectious Diseases Institute, Viruses and Emerging Pathogens Program, The Ohio State University, Columbus, OH, USA.; 3 Department of Microbiology and Immunology, The University of Minnesota, Minneapolis, MN, USA.; 4 Department of Physiology and Cell Biology, The Ohio State University, Columbus, OH, USA.; 5 Department of Surgery, The Ohio State University, Columbus, OH, USA.

## Abstract

Cardiac dysfunction is a common complication of severe influenza virus infection, but whether this occurs due to direct infection of cardiac tissue or indirectly through systemic lung inflammation remains unclear. To test the etiology of this aspect of influenza disease, we generated a novel recombinant heart-attenuated influenza virus via genome incorporation of target sequences for miRNAs expressed in cardiomyocytes. Compared with control virus, mice infected with miR-targeted virus had significantly reduced heart viral titers, confirming cardiac attenuation of viral replication. However, this virus was fully replicative in the lungs and induced similar systemic inflammation and weight loss compared to control virus. The miR-targeted virus induced fewer cardiac conduction irregularities and significantly less fibrosis in mice lacking interferon-induced transmembrane protein 3 (IFITM3), which serve as a model for influenza-associated cardiac pathology. We conclude that robust virus replication in the heart is required for pathology, even when lung inflammation is severe.

## Teaser

Direct heart infection, not systemic inflammation, drives influenza virus–associated cardiac pathology in IFITM3 KO mice.

## INTRODUCTION

Seasonal influenza virus remains a major contributor to human mortality, and the potential for emergence of new pandemic strains is an ever-present worldwide concern (*1*–*3*). In addition to the lung damage traditionally associated with these infections, influenza virus can also cause or exacerbate cardiac dysfunction (*4*–*10*). Ample evidence exists for the role of cardiac dysfunction in influenza-associated morbidity and mortality, including the following: (i) Myocarditis is observed in a substantial portion of hospitalized influenza patients (*11*–*13*), (ii) heart damage at autopsy has been reported for fatal seasonal influenza cases (*13*–*16*), (iii) severe cardiac damage was described in nearly all patients who died from infection with the 1918 pandemic influenza virus (*17*), and (iv) cardiac events increase annually during flu season, especially among the unvaccinated (*18*, *19*). Despite the implications for public health, little is known about the underlying mechanisms by which influenza virus causes heart pathology (*11*–*16*).

There is a debate within the clinical literature as to whether influenza virus directly or indirectly causes cardiac complications (*6*–*11*). Although live virus has been detected in human and nonhuman primate heart samples, direct infection of the heart has rarely been investigated (*20*–*24*). Instead, current dogma states that severely infected lungs produce a cytokine storm with systemic cardiotoxic inflammation, which indirectly drives cardiac dysfunction (*6*–*11*, *25*). Attempts to resolve this fundamental question have been hindered by the lack of tractable animal model systems for influenza-mediated cardiac pathology (*26*, *27*).

Laboratory mouse strains generally show minimal cardiac dysfunction upon influenza virus infection, even with high doses of virus (*26*–*28*). Overcoming this obstacle, we recently reported that mice lacking the interferon-induced transmembrane protein 3 (IFITM3) suffer from severe cardiac electrical dysfunction and fibrosis upon influenza virus infection, thus providing a long-sought model for influenza-associated cardiac complications (*29*). IFITM3 is an innate immunity protein that blocks the fusion of viruses with cell membranes, and deficiencies in IFITM3 are among the only known genetic risk factors in humans for developing severe influenza (*30*–*36*). We observed that severe influenza virus–induced cardiac pathology in IFITM3 knockout (KO) mice correlates with markedly increased and sustained viral loads in heart tissue when compared with rapid virus clearance in wild-type (WT) mice (*29*). These results suggested a direct role for influenza virus replication in the heart in driving cardiac dysfunction, but the observed cardiac phenomena could not be decoupled from the severe lung infection and heightened inflammation that also occurs in IFITM3 KO mice (*29*, *32*, *37*).

Here, we sought to address the fundamental question of whether severe lung infection is sufficient to drive influenza-associated cardiac dysfunction, or whether virus replication in heart cells is required. To decouple lung inflammation and the direct infection of cardiomyocytes that both occur in IFITM3 KO mice, we designed, rescued, and validated a recombinant influenza virus that is attenuated for replication in cardiomyocytes while being fully replication competent and inflammatory in the lungs. We accomplished this cardiomyocyte attenuation via incorporation of target sites for muscle-specific microRNAs (miRNAs) miR133b and miR206 into the viral genome in a manner similar to previous engineering of a virus with blunted replication specifically in hematopoietic cells (*38*–*44*). miRNAs are short (<25 nucleotides), noncoding RNAs that interact with complementary sequences of target RNAs to suppress their translation or target them for degradation (*45*, *46*). While some miRNAs are expressed ubiquitously, others are limited to specific tissue or cell types, which allows for tissue-specific gene silencing (*45*, *46*). Using this novel heart-attenuated influenza virus, we found that severe lung inflammation during influenza virus infection, even in highly infected IFITM3 KO mice, was not sufficient to drive cardiac dysfunction in the absence of virus replication in cardiomyocytes. Thus, direct infection and replication of influenza virus in cardiomyocytes is a primary determinant of cardiac pathology associated with severe influenza.

## RESULTS

### Generation of influenza virus with cardiomyocyte-specific attenuation

**Fig. 1.**
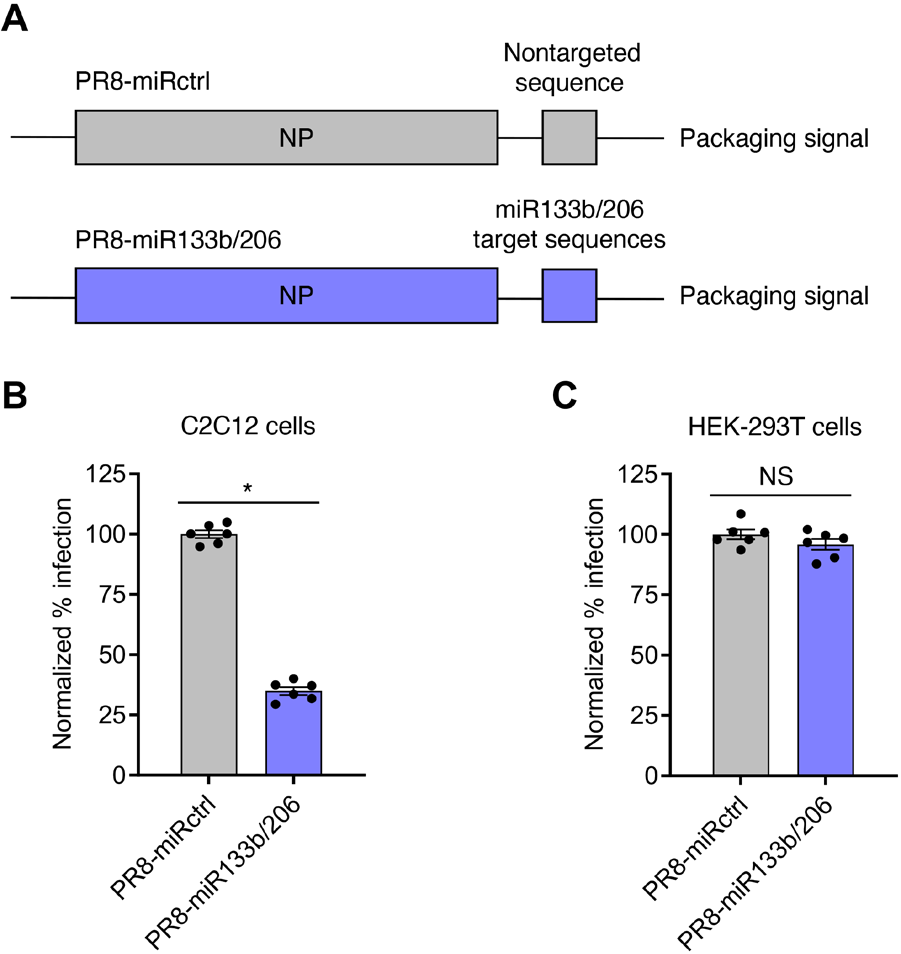
PR8-miR133b/206 is attenuated in myoblast cells in vitro. (**A**) Schematic of miRNA-targeting strategy. Target sequences of two miRNAs expressed in cardiac cells, miR133b and miR206, or a length-matched random sequence were inserted into the influenza A PR8 NP gene, along with a duplicated NP packaging sequence, to generate replication-competent virus with heart-specific attenuation (PR8-miR133b/206) or control virus (PR8-miRctrl). (**B**) C2C12 cells or (**C**) HEK-293T cells were infected with PR8-miR133b/206 or PR8-miRctrl for 24 hours at a multiplicity of infection of 2.5, and percent infection was determined by flow cytometry. Graphs represent normalized infection values. *P < 0.05 by unpaired *t* test; NS, not significant.

To disentangle systemic lung inflammation from cardiac infection in IFITM3 KO mice, we sought to selectively attenuate influenza virus in cardiomyocytes. Influenza virus strain A/Puerto Rico/8/1934 (H1N1) (PR8) is a pathogenic mouse-adapted virus that we previously showed disseminates from the lungs to the hearts of WT and IFITM3 KO mice (*29*). Hence, we chose this virus for an miRNA-based heart-specific attenuation strategy. Using a previously established miRNA-targeting strategy in conjunction with reverse genetics techniques, we inserted into the influenza virus nucleoprotein (NP) gene segment two copies each of target sequences for miR133b and miR206, two miRNAs that are expressed specifically in muscle cells, including cardiomyocytes, and that were used in a previous study to achieve cardiac attenuation of coxsackievirus B3 (Fig. 1A) (*38*–*44*). The NP gene segment was chosen for target site insertion because of its high amino acid conservation among circulating virus strains as well as its relatively high RNA plasticity that together allow recombinant viruses to be generated while limiting reversion mutants (*39*). Likewise, we included two copies of each of the two distinct miRNA target sites to limit effects of any individual point mutation that might occur during viral replication. Following miRNA target site insertions, we added a duplicated NP packaging sequence flanking the inserted target sequence. We rescued this novel recombinant virus in cell culture and herein refer to it as PR8-miR133b/206. A control virus (PR8-miRctrl) containing a length-matched, nontargeted sequence at the same NP gene segment insertion site was described previously (Fig. 1A) (*40*). Both engineered viruses grew to similar high titers [>10^7^ median tissue culture infectious dose (TCID_50_)/ml] in embryonated chicken eggs, indicating that relative replicative capacities of the engineered viruses were unaffected in the absence of specific miRNA targeting.

To validate that PR8-miR133b/206 is attenuated in cells expressing the relevant miRNAs, we infected a mouse myoblast cell line known as C2C12. Compared to the control virus, PR8-miR133b/206 was significantly attenuated in C2C12 cells, suggesting that targeting by miRNAs 133b and 206 potently restricts infection of myoblasts (Fig. 1B). As a control, we observed no significant difference in infection by the two viruses in human embryonic kidney (HEK)–293T cells, which do not express murine miR133b/206 (Fig. 1C). Overall, we established PR8-miR133b/206 as an infectious, replication-competent virus that is attenuated in myocyte-like cells.

### Cardiomyocyte-specific miRNA targeting of influenza virus prolongs survival of IFITM3 KO mice

**Fig. 2.**
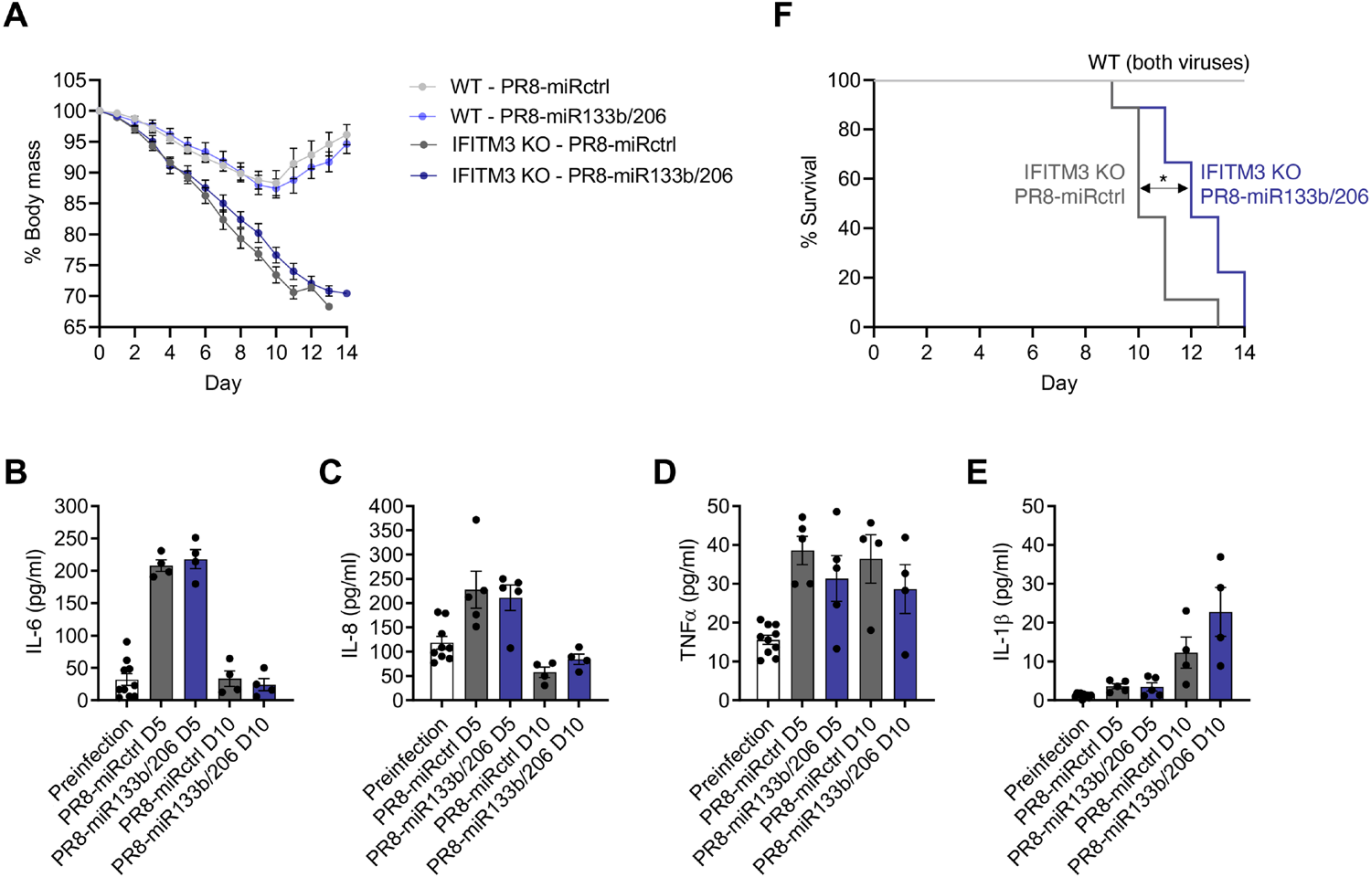
Attenuation of influenza virus cardiac infection does not significantly alter morbidity but decreases mean survival time in IFITM3 KO mice. (**A**) WT and IFITM3 KO mice were intranasally infected with PR8-miR133b/206 or PR8-miRctrl (50 TCID_50_) and monitored daily for weight loss. Points depict mean values collected from at least three experiments, and error bars represent SD of the mean. Differences between WT and KO mouse weights were significant from day 4 onward with *P* < 0.05 by analysis of variance (ANOVA) with Tukey’s multiple comparison test. Differences in weight loss when comparing PR8-miRctrl and PR8-miR133b/206 within the individual mouse genotypes were not significant. (**B** to **E**) A separate cohort of IFITM3 KO mice was infected with PR8-miR133b/206 or PR8-miRctrl (50 TCID_50_). Serum was collected before infection and at days 5 and 10 after infection for multiplex quantification of (B) IL-6, (C) IL-8, (D) TNFα, and (E) IL-1β. Data points represent individual mice, and bars represent mean values. Error bars depict SD of the mean. Comparisons were analyzed by ANOVA followed by Tukey’s post hoc test. No statistically significant differences were observed between the two viruses at either time point. (**F**) Survival curves of mice as infected in (A). The indicated *P* value is for statistical comparison of the IFITM3 KO survival curves (shown by double arrow) as calculated using a Gehan-Breslow-Wilcoxon test.

To measure the overall pathogenicity of PR8-miR133b/206 compared to control virus, we infected WT and IFITM3 KO mice and tracked their weight loss and survival. Consistent with enhanced disease severity in IFITM3 KO mice, the KO animals lost significantly more weight than WT mice in infections with both viruses (Fig. 2A). Comparing the viruses within the individual mouse genotypes, we found that miR133b/206 targeting did not significantly alter the ability of influenza virus to induce weight loss (Fig. 2A). Since weight loss during influenza virus infection is generally driven by cytokine-induced inappetence (*47*–*49*), these data suggest that similar levels of lung-derived inflammation were induced by the two viruses. To test this, we infected an additional cohort of IFITM3 KO mice with PR8-miRctrl or PR8-miR133b/206 and examined systemic inflammation via multiplex enzyme-linked immunosorbent assay (ELISA) measurements of serum cytokine levels. We found that serum levels of canonical proinflammatory cytokines interleukin-6 (IL-6), IL-8, tumor necrosis factor–α (TNFα), and IL-1β were similar when comparing infections with the two viruses (Fig. 2, B to E).

Similarly, all WT mice recovered from infections with either virus strain, while both infections were lethal in IFITM3 KO mice (Fig. 2F). Despite similar weight loss in IFITM3 KO mice, infection with PR8-miR133b/206 resulted in a modest, statistically significant benefit in terms of survival as compared to infection with PR8-miRctrl (median survival times of 10 days for PR8-miRctrl versus 12 days for PR8-miR133b/206) (Fig. 2F). These data suggest that viral replication in cardiomyocytes may contribute to more rapid lethality in IFITM3 KO mice but, not surprisingly, that cardiomyocyte infection is not the sole cause of death. Overall, these outcome data are consistent with the premise that our recombinant viruses can decouple the impact of lung inflammation from the development of influenza-associated cardiac dysfunction.

### PR8-miR133b/206 is specifically attenuated in the heart in vivo

**Fig. 3.**
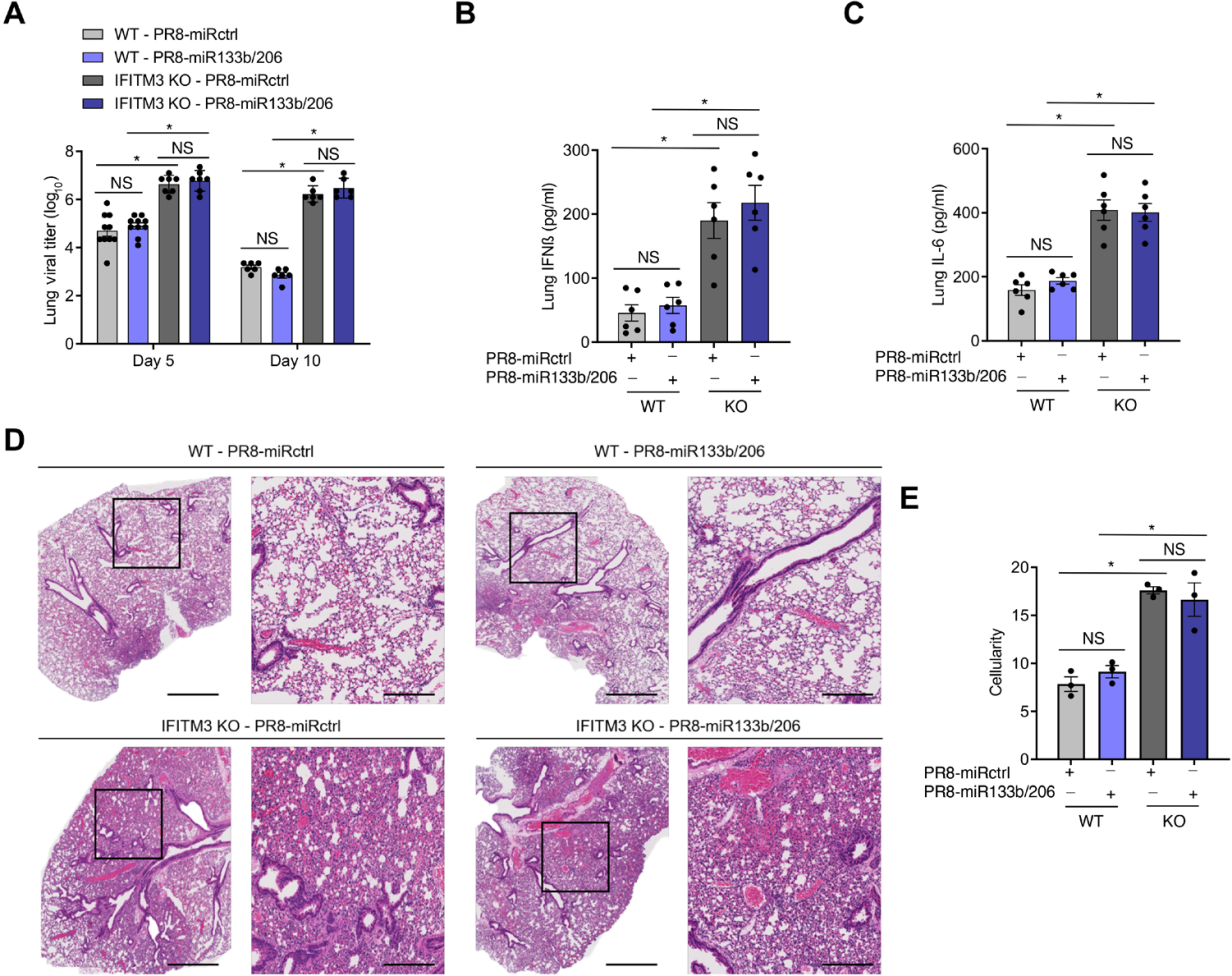
PR8-miR133b/206 is fully pathogenic in the lungs in vivo. WT and IFITM3 KO mice were intranasally infected with PR8-miR133b/206 or PR8-miRctrl (50 TCID_50_). (**A** to **C**) Mice were euthanized on day 5 or 10 after infection for measurement of virus titers (A) or ELISA quantification of IFNβ (B) and IL-6 (C) in the heart. Data points represent individual mice, and bars represent mean values. Error bars depict SD of the mean. Data points are from three independent experiments. Comparisons were analyzed by ANOVA followed by Tukey’s post hoc test. *P < 0.05. (**D**) Mice were euthanized on day 10 after infection for histological analysis of lung pathology. Boxed regions in the left image correspond to the magnified area depicted in the right image for each group. Scale bars, 1 mm and 400 μm for the left and right images, respectively. (**E**) Whole lung images as in (D) were quantified for overall airspace versus cells and other congestion using ImageJ. Data points represent individual mouse lung images, and bars represent mean values. Error bars depict SD of the mean. Comparisons were analyzed by ANOVA followed by Tukey’s post hoc test. *P < 0.05.

To further validate the utility of our engineered viruses in dissecting determinants for cardiac dysfunction, we first examined viral loads and lung inflammation after infection by PR8-miR133b/206 and PR8-miRctrl. As shown in Fig. 3A, lung replication of PR8-miR133b/206 was comparable to that of PR8-miRctrl at both days 5 and 10 after infection in WT mice. As expected, viral titers were higher in IFITM3 KO mice than in WT mice (Fig. 3A), but again were similar in the lungs when comparing PR8-miRctrl versus PR8-miR133b/206 (Fig. 3A). To further confirm that PR8-miR133b/206 lung infections were not attenuated, we measured interferon-β (IFNβ) and IL-6 levels in lung homogenates and found no significant difference for these proinflammatory cytokines in the lungs of mice infected by control or miR-targeted virus (Fig. 3, B and C). We also observed that IFITM3 KO mice, as expected, showed more severe lung histopathology than WT mice (Fig. 3D). Both viruses induced cellular consolidation of the airways at indistinguishable levels (Fig. 3E). Coupled with outcome data (Fig. 2, A and B), the comparable lung pathology and inflammation demonstrate that the novel PR8-miR133b/206 virus is not attenuated in the lungs.

**Fig. 4.**
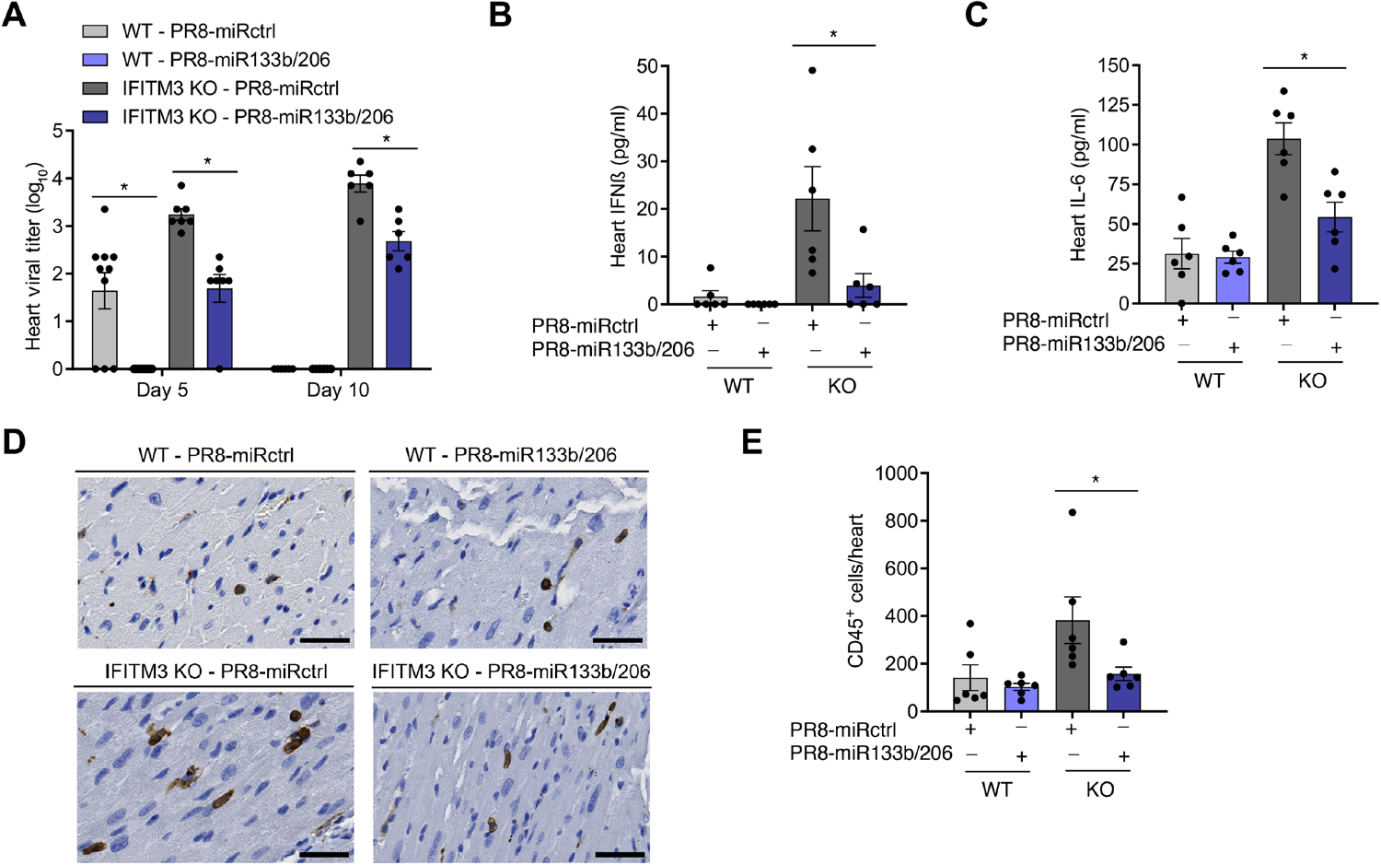
PR8-miR133b/206 is attenuated in the heart in vivo. WT and IFITM3 KO mice were intranasally infected with PR8-miR133b/206 or PR8-miRctrl (50 TCID_50_). (**A** to **C**) Mice were euthanized on day 5 or 10 after infection for TCID_50_ measurement of virus titers (D) or ELISA quantification of IFNβ (B) and IL-6 (C) and in the heart. Data points represent individual mice, and bars represent mean values. Error bars depict SD of the mean. Data points are from three independent experiments. Statistical comparisons were analyzed by ANOVA followed by Tukey’s post hoc test. *P < 0.05. (**D**) Mice were euthanized on day 10 after infection for histological analysis of CD45^+^ immune cell infiltration in the heart. Images shown depict areas of immune cell infiltration indicated by brown staining. Scale bars, 50 μm. (**E**) Whole-heart images were quantified for CD45^+^ cells using ImageJ. Data points represent individual mouse heart images, and bars represent mean values. Error bars depict SD of the mean. Comparisons were analyzed by ANOVA followed by Tukey’s post hoc test. *P < 0.05.

Given the inclusion of muscle/cardiomyocyte-specific miRNA-targeting sequences, we predicted that PR8-miR133b/206 infections would be attenuated in cardiac tissues. To test this prediction, we quantified virus titers in the same experimental mice used to derive the data from lungs (5 and 10 days after infection). As expected for PR8-miRctrl, we observed primarily low or undetectable viral titers in WT mouse hearts and significantly higher titers in the cardiac tissue from IFITM3 KO mice at both time points (Fig. 4A). In PR8-miR133b/206 samples, live virus was undetectable in WT hearts, and the mean titers were significantly lower in IFITM3 KO hearts compared to control virus, confirming overall attenuation of replication in the heart for the miR-targeted virus (Fig. 4A). Cardiomyocyte-specific attenuation of the virus via miR targeting revealed an important role for the direct infection of cardiomyocytes in influenza-associated cardiac inflammation, as manifested in IFITM3 KO mice by (i) a roughly 1- to 1.5-log decrease in mean cardiac viral titers, (ii) markedly reduced levels of inflammatory cytokines IFNβ and IL-6 (Fig. 4, B and C), and (iii) attenuated CD45-positive immune cell infiltration (Fig. 4, D and E).

Overall, we have established a controlled experimental system to interrogate roles of lung inflammation versus direct cardiac infection in influenza-associated cardiac dysfunction. Namely, IFITM3 KO mice infected with PR8-miRctrl or PR8-miR133b/206 allow direct comparison of heart phenotypes in animals with equivalently severe lung infections, but with or without high virus replication in the heart.

### Virus replication in the heart is required for robust induction of cardiac fibrosis and electrical dysfunction

**Fig. 5.**
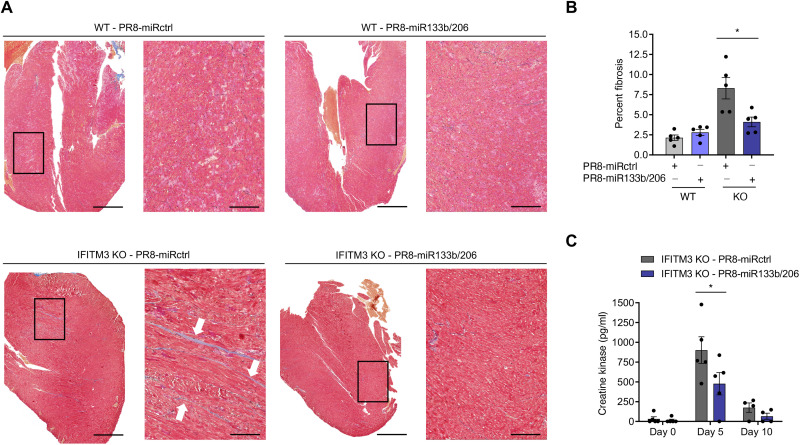
Virus replication in the heart is necessary to induce fibrosis during infection. WT and IFITM3 KO mice were intranasally infected with PR8-miR133b/206 or PR8-miRctrl (50 TCID_50_). (**A**) Hearts were collected on day 10 after infection, and sections were stained with Masson’s trichrome stain, in which blue staining is indicative of fibrotic collagen deposition. Histological processing and image acquisition were performed by the OSU Comparative Pathology and Mouse Phenotyping Core Facility on heart tissue samples provided by A.D.K. A representative heart section is shown for each genotype-virus combination. Boxed areas are regions magnified in the far-right images. Scale bars, 1 mm and 200 μm for the left and right images, respectively. (**B**) Percent fibrosis was calculated by quantifying ratio of blue pixel intensity to total pixel intensity for each heart section. Each point represents a heart from an individual mouse, and bars represent mean values. Error bars represent SD of the mean. Comparisons were analyzed by ANOVA followed by Tukey’s post hoc test. *P < 0.05. (**C**) Serum from IFITM3 KO mice was collected before infection and at days 5 and 10 after infection with PR8-miRctrl or PR8-miR133b/206 for ELISA quantification of creatine kinase. Data points represent individual mice, and bars represent mean values. Error bars depict SD of the mean. Comparisons were analyzed by ANOVA followed by Tukey’s post hoc test. *P < 0.05.

Fibrosis is a broadly observed consequence of severe infectious insults to cardiac tissue in humans. Because IFITM3 KO mice exhibit significant cardiac fibrosis following infection with influenza virus (*29*), and because lung inflammation is not attenuated for our heart-attenuated virus, we could test whether cardiac pathology requires robust heart infection or is induced indirectly by severe lung inflammation. We thus collected hearts from WT and IFITM3 KO mice at day 10 after infection and first performed histological analysis of fibrosis using Masson’s trichrome staining. Examination of heart sections revealed blue-stained fibrotic lesions that were most apparent in hearts from IFITM3 KO mice infected with PR8-miRctrl (Fig. 5A). Fibrotic lesions in WT samples and those from IFITM3 KO mice infected with PR8-miR133b/206 were minimal (Fig. 5A). Quantitative analysis of images from multiple mice confirmed that fibrotic staining in heart samples from IFITM3 KO mice infected with miR-targeted virus was significantly decreased compared to infection with miRctrl virus (Fig. 5B). We also investigated circulating signs of cardiac damage in a cohort of IFITM3 KO mice by measuring secretion of the heart-specific isoenzyme of creatine kinase (CK-MB) into the blood and found that this biomarker of myocardial cell injury was reduced in mice infected with PR8-miR133b/206 as compared to control virus (Fig. 5C) (*50*, *51*). Thus, attenuation of virus replication in the heart correlates with less cardiac muscle damage and fibrosis following infection, indicating that direct virus replication in cardiomyocytes is required for the development of influenza-associated cardiac pathology.

**Fig. 6.**
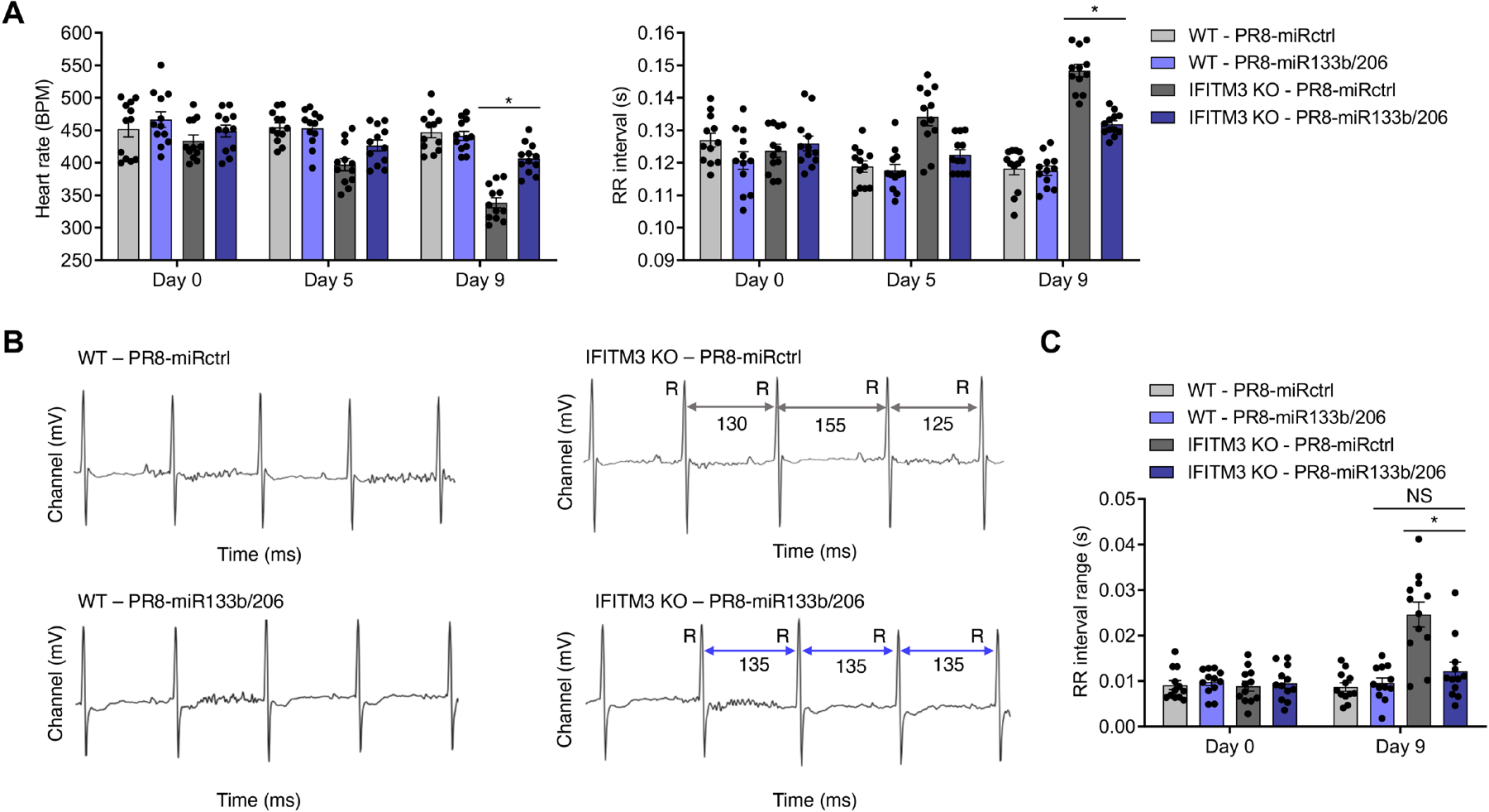
Virus replication in the heart drives cardiac dysfunction during infection. WT and IFITM3 KO mice were intranasally infected with PR8-miR133b/206 or PR8-miRctrl (50 TCID_50_). (**A**) ECG measurements over the time course of infection. Data were collected over at least three independent experiments. Each point represents an individual mouse, and bars represent mean values. Error bars represent SD of the mean. Comparisons were analyzed by ANOVA followed by Tukey’s post hoc test. *P < 0.05. (**B**) Example ECG readings from each genotype-virus combination. Selected RR intervals of the infected KO mice are highlighted by gray (PR8-miRctrl) or purple (PR8-miR133b/206) double arrows. (**C**) RR interval ranges, defined as the difference between the longest and shortest RR intervals over an ECG measurement period of 5 min, were calculated for individual mice on day 9 after infection. Each point represents an individual mouse, and bars represent mean values. Error bars represent SD of the mean. Comparisons were analyzed by ANOVA followed by Tukey’s post hoc test. *P < 0.05. BPM, beats per minute.

Given that cardiac fibrosis is a well-established risk factor for cardiac electrical conduction irregularities (*52*, *53*), and that fibrosis was significantly decreased in infection with PR8-miR133b/206 (Fig. 5), we tested whether cardiac electrical dysfunction induced by influenza virus similarly requires direct infection of cardiomyocytes. We performed electrocardiogram (ECG) measurements on WT and IFITM3 KO mice before infection and at several time points after infection with PR8-miRctrl or PR8-miR133b/206. While cardiac function in WT mice was largely unchanged throughout infection, IFITM3 KO mice showed depressed heart rates during the course of infection with PR8-miRctrl (Fig. 6A). Likewise, RR intervals, a measurement of time between the major peaks (R peaks) on the ECG tracings and which are inversely correlated with heart rate, were commensurately elevated in IFITM3 KO mice infected with PR8-miRctrl (Fig. 6A). We also observed irregular ECG tracings in the KO animals infected with PR8-miRctrl, as defined by irregularly timed subsequent RR intervals (Fig. 6B). This phenotype was largely absent in most of the IFITM3 KO mice infected with PR8-miR133b/206 (Fig. 6B). Specifically, RR interval ranges (defined as the longest RR interval minus the shortest RR interval) (*29*) calculated from 5-min ECG recordings in multiple mice were significantly lower in KO mice infected with PR8-miR133b/206 compared to PR8-miRctrl (Fig. 6C). Although variability in these values can arise from a diverse set of factors, the observed divergence from baseline values of noninfected mice and marked beat-to-beat differences in conjunction with heart rate depression suggest pathological changes in the cardiac function of IFITM3 KO mice following infection with PR8-miRctrl (Fig. 6, B and C) (*54*). Overall, the attenuation in heart viral titers observed for infections with PR8-miR133b/206 is accompanied by decreased cardiac electrical dysfunction, despite the robust virus replication and inflammation in the lungs. We conclude that influenza-associated cardiac fibrosis and electrical dysfunction require direct virus replication in the heart.

## DISCUSSION

Cardiac manifestations of influenza virus infection are widely attributed to severe lung inflammation, which contributes to systemic tissue damage and exacerbates preexisting heart conditions (*55*–*57*). However, given that we lack noninvasive clinical tests to identify direct heart infection by influenza virus in living humans, it has been difficult to determine the relative contributions of lung inflammation versus direct virus-induced damage to the heart in cardiac dysfunction during severe infection. To address the fundamental question of whether severe lung and systemic inflammation alone is sufficient to induce cardiac dysfunction, we turned to an animal model, specifically IFITM3 KO mice, which serve as a severe infection model to study influenza-induced cardiac dysfunction (*29*). IFITM3 alters cell membranes to disfavor virus-to-cell fusion (*58*–*62*). IFITM3 KO mice thus experience increased cellular infection and viral spread in the lung, spleen, and heart (*29*), organs that are naturally susceptible to infection in WT mice. Viremic infection of other organs—such as the brain, liver, or kidneys—is not observed in IFITM3 KO mice (*29*), thus providing a severe infection model recapitulating the tissue-specific distribution of influenza virus dissemination. Further supporting its relevance in dissecting influenza pathologies, genetic deficiencies in IFITM3 associate with susceptibility to severe disease in humans (*30*–*36*).

To manipulate the ability of influenza virus to replicate in cardiomyocytes, we generated a novel recombinant virus strain with cardiomyocyte-specific miRNA target sites. We found that insertion of miRNA target sites for miR133b and miR206 into the NP genome segment of influenza A virus strain PR8 effectively attenuated infection specifically in murine myocyte-like cells in vitro and in the heart in vivo (Figs. 1 and 4). The reduction of virus load in the heart correlated with less severe cardiac fibrosis, inflammation, circulating creatine kinase levels, and electrical dysfunction, although lung virus replication and inflammation, as well as systemic inflammation, remained robust and comparable to control virus (Figs. 3, 5, and 6). Thus, we identified that direct infection of heart cells is required for cardiac dysfunction during influenza virus infection in IFITM3 KO mice. Our findings refute the notion that severe lung inflammation alone is sufficient for influenza-associated cardiac pathologies, although it is plausible that systemic inflammation may compound effects of direct cardiac infection. Since cardiac complications of severe influenza are often seen in hospitalized patients (*11*–*13*), our results may suggest that direct infection of the human heart is more common than currently appreciated. Continued study of both direct cardiac infection and lung pathology and inflammation in cardiac dysfunction, as well as their potential synchronous functions in the clinical setting, is warranted.

Both cardiac fibrosis and cardiac electric dysfunction were reduced in IFITM3 KO mice infected with PR8-miR133b/206 as compared to control virus, despite retention of residual virus in the heart (Figs. 5 and 6). This residual virus replication may be due to incomplete viral attenuation in cardiomyocytes or to replication in other cell types that lack expression of miR133b or miR206. Nonetheless, these data suggest that a threshold of influenza virus in heart tissue is tolerated without producing significant pathology. This notion is strengthened by the fact that WT mice often have low, quickly cleared levels of influenza virus in the heart at early time points after infection but rarely exhibit significant cardiac dysfunction (*27*–*29*). Alternatively, infection of cell types in the heart, other than cardiomyocytes, may occur without major pathological outcomes. However, we observed no protective advantage of reduced cardiac infection in IFITM3 KO mice in terms of weight loss (Fig. 2), a finding that underscores the severity of the lung infection and the accompanying systemic inflammation experienced by IFITM3 KO mice.

Several key issues remain to be addressed by future approaches in dissecting cardiac pathogenesis of influenza virus. Of particular interest is the mechanism by which the virus spreads from the primary site of infection (respiratory tract/lungs) to the heart and how productive infection in the heart is achieved thereafter. Single-cell analyses may prove useful for identifying cell subsets in the heart that are infected initially by influenza virus. In addition, virus strain–specific differences influencing not only overall virulence but also tissue tropism may influence cardiac infection and pathology. Certain strains of influenza virus have been shown to preferentially infect upper versus lower respiratory tract, and other sites of extrapulmonary tropism are noted for particular virus strains (*63*–*67*). Identifying viral factors that influence cardiac infection could prove crucial for predicting and treating cardiac manifestations of both circulating and emerging viruses. Last, there is much to be learned about the clinical role of cardiac infection in humans, particularly in individuals with deleterious *IFITM3* single-nucleotide polymorphisms, who may have a greater risk for direct influenza virus infection of the heart and cardiac pathology. Overall, extrapulmonary manifestations of respiratory virus infections are increasingly appreciated as important aspects of disease that will require continued research. Understanding the direct and indirect effects of respiratory viruses in extrapulmonary tissues, such as the direct effects of influenza virus on the heart as uncovered here, will be critical for combating these noncanonical disease pathologies.

## MATERIALS AND METHODS

### Virus generation, propagation, and titering

PR8-miRctrl and PR8-miR133b/206 were generated as previously described (*38*–*44*). Briefly, two copies each of target sequences for miR133b and miR206, or a length-matched untargeted sequence, were cloned and ligated into the 3′ untranslated region of the NP gene along with a duplicated 3′ NP packaging sequence. The recombinant viruses with modified NP segments were rescued using reverse genetic techniques and plaque-purified. Viruses were propagated in 10-day-old embryonated chicken eggs (Charles River Laboratories) for 48 hours at 37°C and titered on Madin-Darby canine kidney (MDCK) cells. For determining organ titers, tissues were collected and homogenized in 500 μl of phosphate-buffered saline (PBS), flash-frozen, and stored at −80°C before titering on MDCK cells.

### Mouse infections

Mice 8 to12 weeks of age were anesthetized with isoflurane (Henry Schein Animal Health) and intranasally infected with PR8-miRctrl or PR8-miR133b/206 (50 TCID_50_) in sterile saline. Mice were monitored daily for weight loss and morbidity and sacrificed if weight loss exceeded 30% of starting body mass or other end-point criteria (severe hunched posture and lack of ambulation) were met. All procedures were approved by the Ohio State University (OSU) Institutional Animal Care and Use Committee (IACUC).

### Cell lines, cell line infections, and flow cytometry

C2C12, HEK-293T, and MDCK cells were grown in Dulbecco’s modified Eagle’s medium (DMEM) supplemented with 10% EqualFETAL bovine serum (Atlas Biologicals) at 37°C with 5% CO_2_ in a humidified incubator. C2C12 and HEK-293T cells were infected with PR8-miRctrl or PR8-miR133b/206 at a multiplicity of infection of 1.0 for 24 hours. For determination of influenza A virus (IAV) infection percentages via flow cytometry, cells were stained with anti-H1N1 IAV NP (BEI Resources, clone 4F2) and Alexa Fluor 488–conjugated secondary antibody (Thermo Fisher Scientific). Flow cytometry was performed on a FACSCanto II flow cytometer (BD Biosciences) and analyzed using FlowJo software.

### Enzyme-linked immunosorbent assay

IFNβ and IL-6 concentrations in organ homogenates were analyzed using mouse DuoSet ELISA kits (R&D Systems). Quantification of serum IL-6, IL-8, IL-1β, and TNFα was performed using a mouse V-PLEX Proinflammatory Panel 1 kit (Meso Scale Diagnostics) and was analyzed by the OSU Center for Clinical and Translational Science. Creatine kinase quantification was performed using the Mouse Creatine Kinase MB ELISA Kit (Novus Biologicals).

### Electrocardiography

For subsurface ECG recordings, anesthesia was provided by isoflurane in oxygen at a flow rate of 1.0 liter/min. Mice were placed in a prone position on a heated pad to maintain body temperature, and subcutaneous electrodes were placed under the skin (lead II configuration). ECGs were recorded for 5 min on a PowerLab 4/30 (AD Instruments). Anesthesia was maintained for the duration of the reading. Manual visual inspection of ECG tracings for artifacts or abnormalities was performed before data analysis. ECG traces were then analyzed using LabChart 8 Pro software (AD Instruments) wherein the analysis software performed automatic detection of P, Q, R, S, and T wave onsets, amplitudes, and intervals following data recording. The selections generated by the software were then manually optimized, and individual values and averages of 10-s intervals were recorded.

### Immunohistochemistry

For immunohistochemistry, lungs and hearts were fixed in 10% formalin and maintained at 4°C until embedded in paraffin. Organs were sectioned by the OSU Comparative Pathology and Mouse Phenotyping Shared Resource. Masson’s trichrome staining was used to identify fibrotic replacement of cardiac tissue. Lungs were subjected to hematoxylin and eosin staining to measure gross pathology. Digital images of heart sections were generated using Aperio ImageScope software (Leica Biosystems). Fibrosis images were analyzed via ImageJ (version 2.0.0) as previously described (*29*). Lung congestion scores were calculated using a color deconvolution-thresholding method in ImageJ.

## Appendix

View/request a protocol for this paper from *Bio-protocol*.

## References

[1] N. A. Molinari, I. R. Ortega-Sanchez, M. L. Messonnier, W. W. Thompson, P. M. Wortley, E. Weintraub, C. B. Bridges, The annual impact of seasonal influenza in the US: Measuring disease burden and costs. Vaccine 25, 5086–5096 (2007). 17544181 10.1016/j.vaccine.2007.03.046

[2] D. S. Fedson, Influenza, evolution, and the next pandemic. Evol. Med. Public Health 2018, 260–269 (2018). 30455951 10.1093/emph/eoy027 PMC6234328

[3] E. S. Bailey, J. Y. Choi, J. K. Fieldhouse, L. K. Borkenhagen, J. Zemke, D. Zhang, G. C. Gray, The continual threat of influenza virus infections at the human-animal interface: What is new from a one health perspective? Evol. Med. Public Health 2018, 192–198 (2018). 30210800 10.1093/emph/eoy013 PMC6128238

[4] Z. R. Estabragh, M. A. Mamas, The cardiovascular manifestations of influenza: A systematic review. Int. J. Cardiol. 167, 2397–2403 (2013). 23474244 10.1016/j.ijcard.2013.01.274

[5] A. Ukimura, H. Satomi, Y. Ooi, Y. Kanzaki, Myocarditis associated with influenza a H1N1pdm2009. Influenza Res. Treat. 2012, 351979 (2012). 23304476 10.1155/2012/351979 PMC3533457

[6] B. Chacko, J. V. Peter, K. Pichamuthu, K. Ramakrishna, M. Moorthy, R. Karthik, G. John, Cardiac manifestations in patients with pandemic (H1N1) 2009 virus infection needing intensive care. J. Crit. Care 27, 106.e1–106.e6 (2012). 10.1016/j.jcrc.2011.05.016 21737242

[7] V. F. Corrales-Medina, M. Madjid, D. M. Musher, Role of acute infection in triggering acute coronary syndromes. Lancet Infect. Dis. 10, 83–92 (2010). 20113977 10.1016/S1473-3099(09)70331-7

[8] S. A. Sellers, R. S. Hagan, F. G. Hayden, W. A. Fischer II, The hidden burden of influenza: A review of the extra-pulmonary complications of influenza infection. Influenza Other Respi. Viruses 11, 372–393 (2017). 10.1111/irv.12470 PMC5596521 28745014

[9] J. Wang, H. Xu, X. Yang, D. Zhao, S. Liu, X. Sun, J. A. Huang, Q. Guo, Cardiac complications associated with the influenza viruses A subtype H7N9 or pandemic H1N1 in critically ill patients under intensive care. Braz. J. Infect. Dis. 21, 12–18 (2017). 27912070 10.1016/j.bjid.2016.10.005 PMC9425542

[10] E. J. Chow, M. A. Rolfes, A. O’Halloran, E. J. Anderson, N. M. Bennett, L. Billing, S. Chai, E. Dufort, R. Herlihy, S. Kim, R. Lynfield, C. McMullen, M. L. Monroe, W. Schaffner, M. Spencer, H. K. Talbot, A. Thomas, K. Yousey-Hindes, C. Reed, S. Garg, Acute cardiovascular events associated with influenza in hospitalized adults: A cross-sectional smtudy. Ann. Intern. Med. 173, 605–613 (2020). 32833488 10.7326/M20-1509 PMC8097760

[11] M. Kodama, Influenza myocarditis. Circ. J. 74, 2060–2061 (2010). 20838004 10.1253/circj.cj-10-0833

[12] J. Karjalainen, M. S. Nieminen, J. Heikkila, Influenza A1 myocarditis in conscripts. Acta Med. Scand. 207, 27–30 (1980). 7368969 10.1111/j.0954-6820.1980.tb09670.x

[13] A. Ukimura, Y. Ooi, Y. Kanzaki, T. Inomata, T. Izumi, A national survey on myocarditis associated with influenza H1N1pdm2009 in the pandemic and postpandemic season in Japan. J. Infect. Chemother. 19, 426–431 (2013). 23089894 10.1007/s10156-012-0499-z

[14] R. Oseasohn, L. Adelson, M. Kaji, Clinicopathologic study of thirty-three fatal cases of Asian influenza. N. Engl. J. Med. 260, 509–518 (1959). 13632920 10.1056/NEJM195903122601101

[15] C. D. Paddock, L. Liu, A. M. Denison, J. H. Bartlett, R. C. Holman, M. Deleon-Carnes, S. L. Emery, C. P. Drew, W. J. Shieh, T. M. Uyeki, S. R. Zaki, Myocardial injury and bacterial pneumonia contribute to the pathogenesis of fatal influenza B virus infection. J. Infect. Dis. 205, 895–905 (2012). 22291193 10.1093/infdis/jir861

[16] A. Ukimura, T. Izumi, A. Matsumori; Clinical Research Committee on Myocarditis Associated with Influenza A (HN) Pandemic in Japan organized by Japanese Circulation Society, A national survey on myocarditis associated with the 2009 influenza A (H1N1) pandemic in Japan. Circ. J. 74, 2193–2199 (2010). 20697177 10.1253/circj.cj-10-0452

[17] B. Lucke, T. Wight, E. Kime, Pathologic anatomy and bacteriology of influenza: Epidemic of autumn, 1918. Arch. Intern. Med. 24, 154–237 (1919).

[18] J. A. Udell, R. Zawi, D. L. Bhatt, M. Keshtkar-Jahromi, F. Gaughran, A. Phrommintikul, A. Ciszewski, H. Vakili, E. B. Hoffman, M. E. Farkouh, C. P. Cannon, Association between influenza vaccination and cardiovascular outcomes in high-risk patients: A meta-analysis. JAMA 310, 1711–1720 (2013). 24150467 10.1001/jama.2013.279206

[19] L. S. Sperling, M. A. Albert, R. Koppaka, Disparities in influenza vaccination-opportunity to extend cardiovascular prevention to millions of hearts. JAMA Cardiol. 6, 11–12 (2021). 32902564 10.1001/jamacardio.2020.3983

[20] D. Kobasa, S. M. Jones, K. Shinya, J. C. Kash, J. Copps, H. Ebihara, Y. Hatta, J. H. Kim, P. Halfmann, M. Hatta, F. Feldmann, J. B. Alimonti, L. Fernando, Y. Li, M. G. Katze, H. Feldmann, Y. Kawaoka, Aberrant innate immune response in lethal infection of macaques with the 1918 influenza virus. Nature 445, 319–323 (2007). 17230189 10.1038/nature05495

[21] W. Witzleb, H. Witzleb, J. Mehlhorn, M. Sprossig, P. Wutzler, Demonstration of influenza virus A in human heart by semiquantitative virus assay and immunofluorescence. Acta Virol. 20, 168 (1976). 5876

[22] A. M. Cioc, G. J. Nuovo, Histologic and in situ viral findings in the myocardium in cases of sudden, unexpected death. Mod. Pathol. 15, 914–922 (2002). 12218208 10.1097/01.MP.0000024291.37651.CD

[23] C. G. Ray, T. B. Icenogle, L. L. Minnich, J. G. Copeland, T. M. Grogan, The use of intravenous ribavirin to treat influenza virus-associated acute myocarditis. J. Infect. Dis. 159, 829–836 (1989). 2775346 10.1093/infdis/159.5.829

[24] N. E. Bowles, J. Y. Ni, D. L. Kearney, M. Pauschinger, H. P. Schultheiss, R. McCarthy, J. Hare, J. T. Bricker, K. R. Bowles, J. A. Towbin, Detection of viruses in myocardial tissues by polymerase chain reaction: Evidence of adenovirus as a common cause of myocarditis in children and adults. J. Am. Coll. Cardiol. 42, 466–472 (2003). 12906974 10.1016/s0735-1097(03)00648-x

[25] C. Tschope, E. Ammirati, B. Bozkurt, A. L. P. Caforio, L. T. Cooper, S. B. Felix, J. M. Hare, B. Heidecker, S. Heymans, N. Hubner, S. Kelle, K. Klingel, H. Maatz, A. S. Parwani, F. Spillmann, R. C. Starling, H. Tsutsui, P. Seferovic, S. Van Linthout, Myocarditis and inflammatory cardiomyopathy: Current evidence and future directions. Nat. Rev. Cardiol. 18, 169–193 (2021). 33046850 10.1038/s41569-020-00435-x PMC7548534

[26] T. Fislova, M. Gocnik, T. Sladkova, V. Durmanova, J. Rajcani, E. Vareckova, V. Mucha, F. Kostolansky, Multiorgan distribution of human influenza a virus strains observed in a mouse model. Arch. Virol. 154, 409–419 (2009). 19189197 10.1007/s00705-009-0318-8

[27] M. Kotaka, Y. Kitaura, H. Deguchi, K. Kawamura, Experimental influenza A virus myocarditis in mice. Light and electron microscopic, virologic, and hemodynamic study. Am. J. Pathol. 136, 409–419 (1990). 2154929 PMC1877391

[28] D. Filgueiras-Rama, J. Vasilijevic, J. Jalife, S. F. Noujaim, J. M. Alfonso, J. A. Nicolas-Avila, C. Gutierrez, N. Zamarreno, A. Hidalgo, A. Bernabe, C. P. Cop, D. Ponce-Balbuena, G. Guerrero-Serna, D. Calle, M. Desco, J. Ruiz-Cabello, A. Nieto, A. Falcon, Human influenza A virus causes myocardial and cardiac-specific conduction system infections associated with early inflammation and premature death. Cardiovasc. Res. 117, 876–889 (2021). 32346730 10.1093/cvr/cvaa117 PMC7898948

[29] A. D. Kenney, T. M. McMichael, A. Imas, N. M. Chesarino, L. Zhang, L. E. Dorn, Q. Wu, O. Alfaour, F. Amari, M. Chen, A. Zani, M. Chemudupati, F. Accornero, V. Coppola, M. V. S. Rajaram, J. S. Yount, IFITM3 protects the heart during influenza virus infection. Proc. Natl. Acad. Sci. U.S.A. 116, 18607–18612 (2019). 31451661 10.1073/pnas.1900784116 PMC6744864

[30] A. Zani, J. S. Yount, Antiviral protection by IFITM3 in vivo. Curr. Clin. Microbiol. Rep. 5, 229–237 (2018). 30662816 10.1007/s40588-018-0103-0 PMC6334760

[31] A. D. Kenney, J. A. Dowdle, L. Bozzacco, T. M. McMichael, C. S. Gelais, A. R. Panfil, Y. Sun, L. S. Schlesinger, M. Z. Anderson, P. L. Green, C. B. Lopez, B. R. Rosenberg, L. Wu, J. S. Yount, Human genetic determinants of viral diseases. Annu. Rev. Genet. 51, 241–263 (2017). 28853921 10.1146/annurev-genet-120116-023425 PMC6038703

[32] A. R. Everitt, S. Clare, T. Pertel, S. P. John, R. S. Wash, S. E. Smith, C. R. Chin, E. M. Feeley, J. S. Sims, D. J. Adams, H. M. Wise, L. Kane, D. Goulding, P. Digard, V. Anttila, J. K. Baillie, T. S. Walsh, D. A. Hume, A. Palotie, Y. Xue, V. Colonna, C. Tyler-Smith, J. Dunning, S. B. Gordon, I. I. Gen, M. Investigators, R. L. Smyth, P. J. Openshaw, G. Dougan, A. L. Brass, P. Kellam, IFITM3 restricts the morbidity and mortality associated with influenza. Nature 484, 519–523 (2012). 22446628 10.1038/nature10921 PMC3648786

[33] E. K. Allen, A. G. Randolph, T. Bhangale, P. Dogra, M. Ohlson, C. M. Oshansky, A. E. Zamora, J. P. Shannon, D. Finkelstein, A. Dressen, J. DeVincenzo, M. Caniza, B. Youngblood, C. M. Rosenberger, P. G. Thomas, SNP-mediated disruption of CTCF binding at the IFITM3 promoter is associated with risk of severe influenza in humans. Nat. Med. 23, 975–983 (2017). 28714988 10.1038/nm.4370 PMC5702558

[34] Z. Wang, A. Zhang, Y. Wan, X. Liu, C. Qiu, X. Xi, Y. Ren, J. Wang, Y. Dong, M. Bao, L. Li, M. Zhou, S. Yuan, J. Sun, Z. Zhu, L. Chen, Q. Li, Z. Zhang, X. Zhang, S. Lu, P. C. Doherty, K. Kedzierska, J. Xu, Early hypercytokinemia is associated with interferon-induced transmembrane protein-3 dysfunction and predictive of fatal H7N9 infection. Proc. Natl. Acad. Sci. U.S.A. 111, 769–774 (2014). 24367104 10.1073/pnas.1321748111 PMC3896201

[35] N. Lee, B. Cao, C. Ke, H. Lu, Y. Hu, C. H. T. Tam, R. C. W. Ma, D. Guan, Z. Zhu, H. Li, M. Lin, R. Y. K. Wong, I. M. H. Yung, T. N. Hung, K. Kwok, P. Horby, D. S. C. Hui, M. C. W. Chan, P. K. S. Chan, IFITM3, TLR3, and CD55 gene SNPs and cumulative genetic risks for severe outcomes in chinese patients with H7N9/H1N1pdm09 influenza. J. Infect. Dis. 216, 97–104 (2017). 28510725 10.1093/infdis/jix235 PMC7107409

[36] Y. H. Zhang, Y. Zhao, N. Li, Y. C. Peng, E. Giannoulatou, R. H. Jin, H. P. Yan, H. Wu, J. H. Liu, N. Liu, D. Y. Wang, Y. L. Shu, L. P. Ho, P. Kellam, A. McMichael, T. Dong, Interferon-induced transmembrane protein-3 genetic variant rs12252-C is associated with severe influenza in Chinese individuals. Nat. Commun. 4, 1418 (2013). 23361009 10.1038/ncomms2433 PMC3562464

[37] C. C. Bailey, I. C. Huang, C. Kam, M. Farzan, Ifitm3 limits the severity of acute influenza in mice. PLOS Pathog. 8, e1002909 (2012). 22969429 10.1371/journal.ppat.1002909 PMC3435252

[38] B. D. Brown, B. Gentner, A. Cantore, S. Colleoni, M. Amendola, A. Zingale, A. Baccarini, G. Lazzari, C. Galli, L. Naldini, Endogenous microRNA can be broadly exploited to regulate transgene expression according to tissue, lineage and differentiation state. Nat. Biotechnol. 25, 1457–1467 (2007). 18026085 10.1038/nbt1372

[39] J. T. Perez, A. M. Pham, M. H. Lorini, M. A. Chua, J. Steel, B. R. tenOever, MicroRNA-mediated species-specific attenuation of influenza a virus. Nat. Biotechnol. 27, 572–576 (2009). 19483680 10.1038/nbt.1542

[40] R. A. Langlois, A. Varble, M. A. Chua, A. Garcia-Sastre, B. R. tenOeverl, Hematopoietic-specific targeting of influenza A virus reveals replication requirements for induction of antiviral immune responses. Proc. Natl. Acad. Sci. U.S.A. 109, 12117–12122 (2012). 22778433 10.1073/pnas.1206039109 PMC3409765

[41] R. A. Langlois, R. A. Albrecht, B. Kimble, T. Sutton, J. S. Shapiro, C. Finch, M. Angel, M. A. Chua, A. S. Gonzalez-Reiche, K. Xu, D. Perez, A. Garcia-Sastre, B. R. tenOever, MicroRNA-based strategy to mitigate the risk of gain-of-function influenza studies. Nat. Biotechnol. 31, 844–847 (2013). 23934176 10.1038/nbt.2666 PMC3808852

[42] F. He, H. Yao, J. Wang, Z. Xiao, L. Xin, Z. Liu, X. Ma, J. Sun, Q. Jin, Z. Liu, Coxsackievirus B3 engineered to contain microRNA targets for muscle-specific microRNAs displays attenuated cardiotropic virulence in mice. J. Virol. 89, 908–916 (2015). 25339771 10.1128/JVI.02933-14 PMC4300646

[43] E. J. Fay, S. L. Aron, I. A. Stone, B. M. Waring, R. K. Plemper, R. A. Langlois, Engineered small-molecule control of influenza a virus replication. J. Virol. 93, e01677-18 (2019). 30282710 10.1128/JVI.01677-18 PMC6288343

[44] E. Hoffmann, G. Neumann, Y. Kawaoka, G. Hobom, R. G. Webster, A DNA transfection system for generation of influenza A virus from eight plasmids. Proc. Natl. Acad. Sci. U.S.A. 97, 6108–6113 (2000). 10801978 10.1073/pnas.100133697 PMC18566

[45] L. F. R. Gebert, I. J. MacRae, Regulation of microRNA function in animals. Nat. Rev. Mol. Cell Biol. 20, 21–37 (2019). 30108335 10.1038/s41580-018-0045-7 PMC6546304

[46] J. O’Brien, H. Hayder, Y. Zayed, C. Peng, Overview of microRNA biogenesis, mechanisms of actions, and circulation. Front. Endocrinol. 9, 402 (2018). 10.3389/fendo.2018.00402 PMC6085463 30123182

[47] H. T. Groves, S. L. Higham, M. F. Moffatt, M. J. Cox, J. S. Tregoning, Respiratory viral infection alters the gut microbiota by inducing inappetence. MBio 11, e03236-19 (2020). 32071269 10.1128/mBio.03236-19 PMC7029140

[48] A. H. Swiergiel, G. N. Smagin, A. J. Dunn, Influenza virus infection of mice induces anorexia: Comparison with endotoxin and interleukin-1 and the effects of indomethacin. Pharmacol. Biochem. Behav. 57, 389–396 (1997). 9164599 10.1016/s0091-3057(96)00335-8

[49] J. P. Konsman, P. Parnet, R. Dantzer, Cytokine-induced sickness behaviour: Mechanisms and implications. Trends Neurosci. 25, 154–159 (2002). 11852148 10.1016/s0166-2236(00)02088-9

[50] M. Kemp, J. Donovan, H. Higham, J. Hooper, Biochemical markers of myocardial injury. Br. J. Anaesth. 93, 63–73 (2004). 15096441 10.1093/bja/aeh148

[51] K. Bachmaier, J. Mair, F. Offner, C. Pummerer, N. Neu, Serum cardiac troponin T and creatine kinase-MB elevations in murine autoimmune myocarditis. Circulation 92, 1927–1932 (1995). 7671377 10.1161/01.cir.92.7.1927

[52] A. M. Segura, O. H. Frazier, L. M. Buja, Fibrosis and heart failure. Heart Fail. Rev. 19, 173–185 (2014). 23124941 10.1007/s10741-012-9365-4

[53] M. Maanja, B. Wieslander, T. T. Schlegel, L. Bacharova, H. Abu Daya, Y. Fridman, T. C. Wong, E. B. Schelbert, M. Ugander, Diffuse myocardial fibrosis reduces electrocardiographic voltage measures of left ventricular hypertrophy independent of left ventricular mass. J. Am. Heart Assoc. 6, e003795 (2017). 28111363 10.1161/JAHA.116.003795 PMC5523623

[54] M. A. Woo, W. G. Stevenson, D. K. Moser, R. B. Trelease, R. M. Harper, Patterns of beat-to-beat heart rate variability in advanced heart failure. Am. Heart J. 123, 704–710 (1992). 1539521 10.1016/0002-8703(92)90510-3

[55] S. Van Eeden, J. Leipsic, S. F. Paul Man, D. D. Sin, The relationship between lung inflammation and cardiovascular disease. Am. J. Respir. Crit. Care Med. 186, 11–16 (2012). 22538803 10.1164/rccm.201203-0455PP

[56] M. Madjid, M. Naghavi, S. Litovsky, S. W. Casscells, Influenza and cardiovascular disease: A new opportunity for prevention and the need for further studies. Circulation 108, 2730–2736 (2003). 14610013 10.1161/01.CIR.0000102380.47012.92

[57] M. Naghavi, P. Wyde, S. Litovsky, M. Madjid, A. Akhtar, S. Naguib, M. S. Siadaty, S. Sanati, W. Casscells, Influenza infection exerts prominent inflammatory and thrombotic effects on the atherosclerotic plaques of apolipoprotein E-deficient mice. Circulation 107, 762–768 (2003). 12578882 10.1161/01.cir.0000048190.68071.2b

[58] N. M. Chesarino, A. A. Compton, T. M. McMichael, A. D. Kenney, L. Zhang, V. Soewarna, M. Davis, O. Schwartz, J. S. Yount, IFITM3 requires an amphipathic helix for antiviral activity. EMBO Rep. 18, 1740–1751 (2017). 28835547 10.15252/embr.201744100 PMC5623871

[59] T. Y. Lin, C. R. Chin, A. R. Everitt, S. Clare, J. M. Perreira, G. Savidis, A. M. Aker, S. P. John, D. Sarlah, E. M. Carreira, S. J. Elledge, P. Kellam, A. L. Brass, Amphotericin B increases influenza A virus infection by preventing IFITM3-mediated restriction. Cell Rep. 5, 895–908 (2013). 24268777 10.1016/j.celrep.2013.10.033 PMC3898084

[60] T. M. Desai, M. Marin, C. R. Chin, G. Savidis, A. L. Brass, G. B. Melikyan, IFITM3 restricts influenza A virus entry by blocking the formation of fusion pores following virus-endosome hemifusion. PLOS Pathog. 10, e1004048 (2014). 24699674 10.1371/journal.ppat.1004048 PMC3974867

[61] X. Guo, J. Steinkuhler, M. Marin, X. Li, W. Lu, R. Dimova, G. B. Melikyan, Interferon-induced transmembrane protein 3 blocks fusion of diverse enveloped viruses by altering mechanical properties of cell membranes. ACS Nano , 8155–8170 (2021). 33656312 10.1021/acsnano.0c10567 PMC8159881

[62] K. Li, R. M. Markosyan, Y. M. Zheng, O. Golfetto, B. Bungart, M. Li, S. Ding, Y. He, C. Liang, J. C. Lee, E. Gratton, F. S. Cohen, S. L. Liu, IFITM proteins restrict viral membrane hemifusion. PLOS Pathog. 9, e1003124 (2013). 23358889 10.1371/journal.ppat.1003124 PMC3554583

[63] R. W. Chan, S. S. Kang, H. L. Yen, A. C. Li, L. L. Tang, W. C. Yu, K. M. Yuen, I. W. Chan, D. D. Wong, W. W. Lai, D. L. Kwong, A. D. Sihoe, L. L. Poon, Y. Guan, J. M. Nicholls, J. S. Peiris, M. C. Chan, Tissue tropism of swine influenza viruses and reassortants in ex vivo cultures of the human respiratory tract and conjunctiva. J. Virol. 85, 11581–11587 (2011). 21880750 10.1128/JVI.05662-11 PMC3209323

[64] M. C. Chan, R. W. Chan, H. L. Yen, W. C. Yu, K. M. Yuen, L. L. Tang, A. C. Li, S. S. Kang, C. F. Hui, W. W. Lai, A. D. Sihoe, Y. Guan, J. M. Nicholls, J. M. Peiris, Tropism and innate host response of the 2009 pandemic H1N1 influenza virus compared with related swine influenza viruses and reassortants in ex vivo and in vitro cultures of the human respiratory tract and conjunctiva. Influenza Other Respir. Viruses 5, 54–55 (2011). 25653710 10.1111/j.1750-2659.2011.00209.x PMC4313891

[65] J. M. Nicholls, M. C. Chan, W. Y. Chan, H. K. Wong, C. Y. Cheung, D. L. Kwong, M. P. Wong, W. H. Chui, L. L. Poon, S. W. Tsao, Y. Guan, J. S. Peiris, Tropism of avian influenza A (H5N1) in the upper and lower respiratory tract. Nat. Med. 13, 147–149 (2007). 17206149 10.1038/nm1529

[66] S. Tundup, M. Kandasamy, J. T. Perez, N. Mena, J. Steel, T. Nagy, R. A. Albrecht, B. Manicassamy, Endothelial cell tropism is a determinant of H5N1 pathogenesis in mammalian species. PLOS Pathog. 13, e1006270 (2017). 28282445 10.1371/journal.ppat.1006270 PMC5362246

[67] S. Li, J. Schulman, S. Itamura, P. Palese, Glycosylation of neuraminidase determines the neurovirulence of influenza A/WSN/33 virus. J. Virol. 67, 6667–6673 (1993). 8411368 10.1128/jvi.67.11.6667-6673.1993 PMC238105

